# Dual-hemispheric transcranial direct current stimulation (tDCS) over primary motor cortex does not affect movement selection

**DOI:** 10.1371/journal.pone.0226103

**Published:** 2019-12-12

**Authors:** Nivethida Thirugnanasambandam, Felix G. Contreras-Castro, Mark Hallett

**Affiliations:** 1 Human Motor Control Section, National Institute of Neurological Disorders and Stroke, National Institutes of Health, Bethesda, MD, United States of America; 2 Amherst College, Amherst, MA, United States of America; University Medical Center Goettingen, GERMANY

## Abstract

Volition and sense of agency are two primary components of a voluntary or internally generated movement. It has been shown that movement selection cannot be altered without interfering with the sense of volition using single pulse transcranial magnetic stimulation over the primary motor cortex. In the current study, we aimed at examining whether modulating the cortical excitability of the final effector in the voluntary motor pathway—the primary motor cortex, using transcranial direct current stimulation (tDCS) would alter movement selection. Our hypothesis was that anodal tDCS would increase motor cortical excitability and thereby decrease the threshold for movement execution, which could favor selection of the contralateral hand. We recruited 13 healthy adults to perform a movement selection task involving free-choice and externally-cued trials while applying real/sham tDCS in a C3-C4 dual-hemispheric electrode montage. Contrary to our hypothesis, we did not observe any effect of tDCS on movement selection either at the individual or group level. However, our data confirms the strong preference of right-handed individuals for the dominant right hand. We also found higher reaction time for internally generated movement compared to externally triggered movement. We therefore conclude that movement selection cannot be influenced at the level of primary motor cortex and that brain areas upstream of the primary motor cortex in the voluntary motor pathway may be possible targets for influencing movement selection.

## Introduction

**Fig 1:**
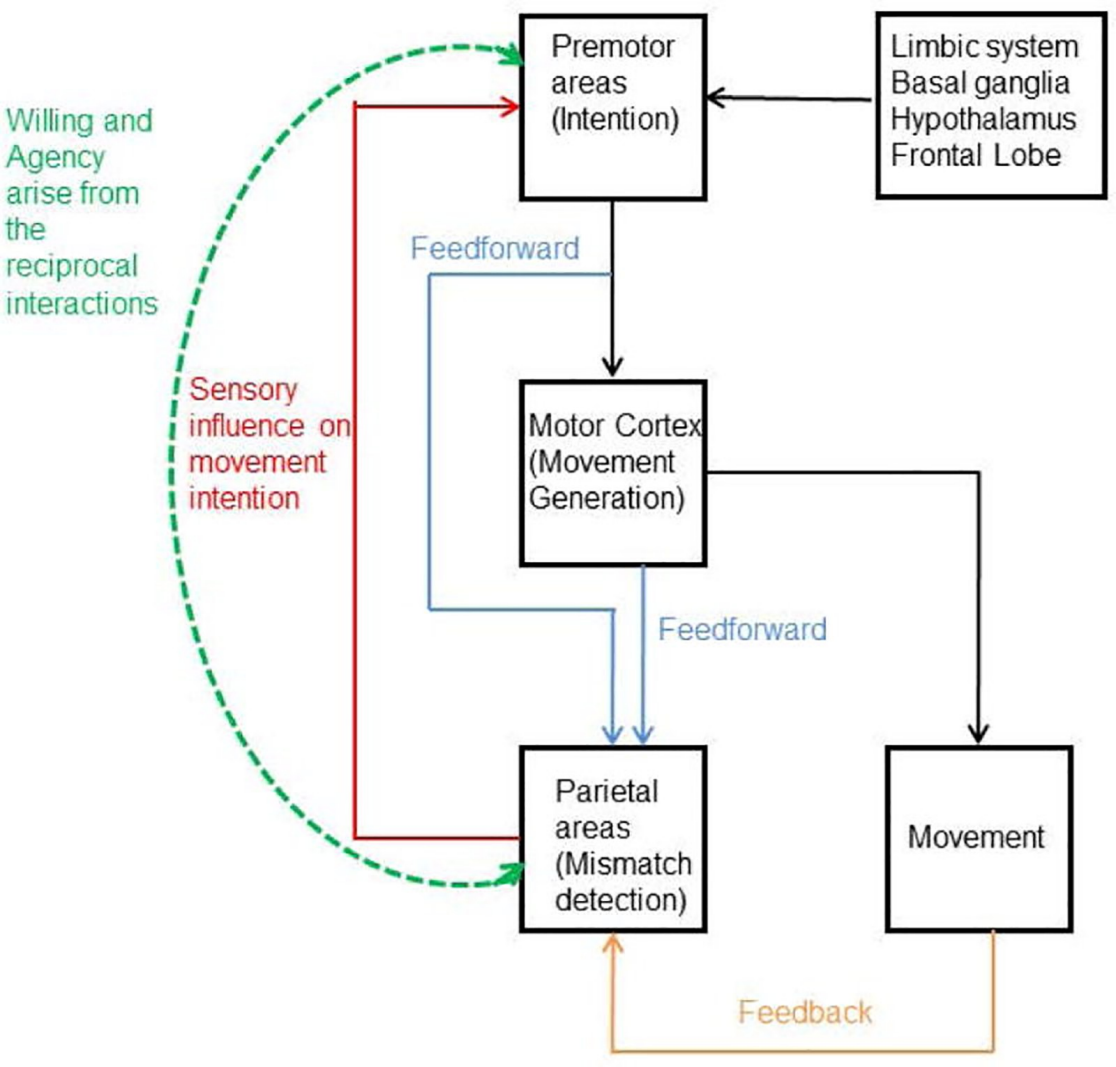
Voluntary motor pathway (reproduced from Hallett, 2016) illustrates the voluntary motor pathway depicting the involvement of the parieto-frontal network in both volition and sense of agency.

Of the several key functions of the brain, the foremost is to produce movement. Movement may be either voluntary or involuntary. What differentiates the two is the sense of “volition” or an intention to move that is associated with the former and consequently a “sense of agency” where one believes that he/she is responsible for the action [1, 2]. The consciousness of volition is fundamental to the experience of healthy adult humans [3]. Voluntary movements can be internally generated or externally triggered. For internally generated movements, motor drive or an intention to move is initiated at the subcortical and prefrontal brain regions, which are further facilitated or inhibited by decisions made at other frontal areas such as the pre-supplementary motor area and dorsolateral prefrontal cortex. Once the decision to move is made, the movement is planned by the premotor cortex, and finally executed by the primary motor cortex. If the execution of the movement results in perceptual feedback from the parietal cortex that matches with the initial motor intention, then sense of agency is generated and the movement is perceived as “voluntary” [1, 4]. This voluntary motor pathway is described in Fig 1 (reproduced from Hallett, 2016). However, our knowledge about the neural processes underlying volition and sense of agency is still limited. Nevertheless, considering its implications in the pathophysiology of neuropsychiatric disorders such as schizophrenia, functional movement disorders, tics and alien hand syndrome, it becomes important to understand the physiology of volition and explore methods to modulate voluntary behavior.

Few studies have investigated if movement selection out of free will can be altered using single pulse transcranial magnetic stimulation (TMS) over the primary motor cortex (M1) [5–7]. They hypothesized that since M1 was responsible for the final execution of the planned voluntary movement, any intervention at the level of M1 should change the pre-planned movement and thereby alter movement selection without affecting the sense of volition. Although the two initial studies demonstrated that it was possible to alter movement selection by a single TMS pulse delivered over M1 [5, 6], later Sohn et al [7] used improved and blinded experimental paradigms to disprove the same. A single TMS pulse might not have been timed appropriately or it did not modulate the excitability of the underlying cortex so as to influence movement selection. The earliest study by Ammon and Gandevia [5] also used low intensity direct current stimulation, but failed to show any effect on movement selection. The field of transcranial direct current stimulation (tDCS) has evolved much since then and we can now say that the intensity used by Ammon and Gandevia was far too small to have been effective. However, their idea to use tDCS to modulate motor cortical excitability to influence movement selection has not been properly investigated.

In the current study, we aimed to investigate the effect of tDCS over the primary motor cortex (M1) on movement selection. For this, we used a simple movement selection task similar to that used in past studies [5–7] and examined the hand preference of healthy adults after applying real or sham tDCS. We hypothesized that anodal tDCS over M1 would increase the cortical excitability of the underlying cortex and thereby enhance the subject’s preference to move the contralateral hand. Our expectation was that anodal tDCS over C3 would increase the underlying cortical excitability and consequently the probability of the subject to move the right hand, which should be reflected as an increase in right hand preference.

## Materials and methods

We recruited 13 healthy adult volunteers (mean age ± SD = 38 ± 13 years; 7 females) for this study from the NINDS database. All subjects were right-handed as assessed by the Edinburg Handedness Inventory [8] and gave written informed consent prior to participation in the study. The study was approved by the institutional review board for NINDS and conformed to the guidelines of the Declaration of Helsinki.

The participants were seated comfortably on a chair with both their elbows flexed to about 90°, forearms pronated and hands resting on a table just in front of them. They were asked to fixate at the center of a computer screen placed approximately a meter away. Surface Ag-AgCl electrodes were placed over bilateral extensor indices muscles to record EMG. EMG data was collected, amplified 1000 times, bandpass filtered (20Hz– 2kHz) using Neuropack-2300 (Nihon Kohden, Japan), digitized using CED micro1401 at a sampling rate of 2KHz and stored for offline analysis using Signal version 5.02 (Cambridge electronic Design, Cambridge, UK).

### Experimental paradigm

The experiment was comprised of 4 blocks of which the polarity of the electrode over C3 in 2 blocks was anodal and 2 blocks was cathodal. The order of the polarity was randomized across subjects. Each block had a set of 96 trials with 64 (two-thirds) free-choice and 32 (one-third) externally-cued trials. Each trial lasted for approximately 6–7.5s. For the externally-cued trials, an arrow appeared at the center of the screen pointing either left or right. The subjects moved the index finger of the hand corresponding to the direction of the arrow. During the free-choice trials, an upward directed arrow appeared on the screen and the subjects were instructed to extend the index finger of either hand at their choice. They were instructed to make a completely random choice of which hand to move and to not follow any specific order or sequence. We also instructed them that they should also try not to make a choice based on their choices in the previous trials. At the end of the experiment, we asked them what strategy they had used to make their choices. Later, during offline analysis, we visually inspected the results to confirm that there was no specific sequence or pattern in the responses, which was the only evidence supporting the idea that the participants’ responses were more or less random.

A pair of wet sponge-covered rubber electrodes (5x5 cm^2^) generating a current density of 6x10^-3^ μA/mm^2^ were placed over bilateral primary motor cortices (C3 and C4 of 10–20 EEG system). One electrode was placed over C3 (left primary motor cortex) and the other electrode was placed over C4 (right primary motor cortex). This dual-hemisphere electrode montage is known to cause greater improvement in motor performance compared to uni-hemisphere stimulation [9]. We used this montage aiming to simultaneously increase cortical excitability of one M1 and decrease that of the other M1, thereby enhancing the expected effect size. A constant current of 1.5mA was applied (neuroConn DC-Stimulator Plus, neuroConn GmbH, Ilmenau, Germany) during the active stimulation trials.

In each block of the experiment, there were equal number of free-choice trials that received sham (32) and real (32) tDCS stimulation. Since we were only interested in determining the impact of tDCS on movement selection, we used only the 64 free-choice trials in every block for analysis. Applying tDCS during externally-cued trials would only have prolonged the duration of the experiment and further decreased the attention levels of the subjects. In order to limit the number of trials, we used the externally-cued trials for ramping up and ramping down the current. The current was ramped up/down over 6s. Thus, no constant current was delivered during externally-cued trials.

**Fig 2:**
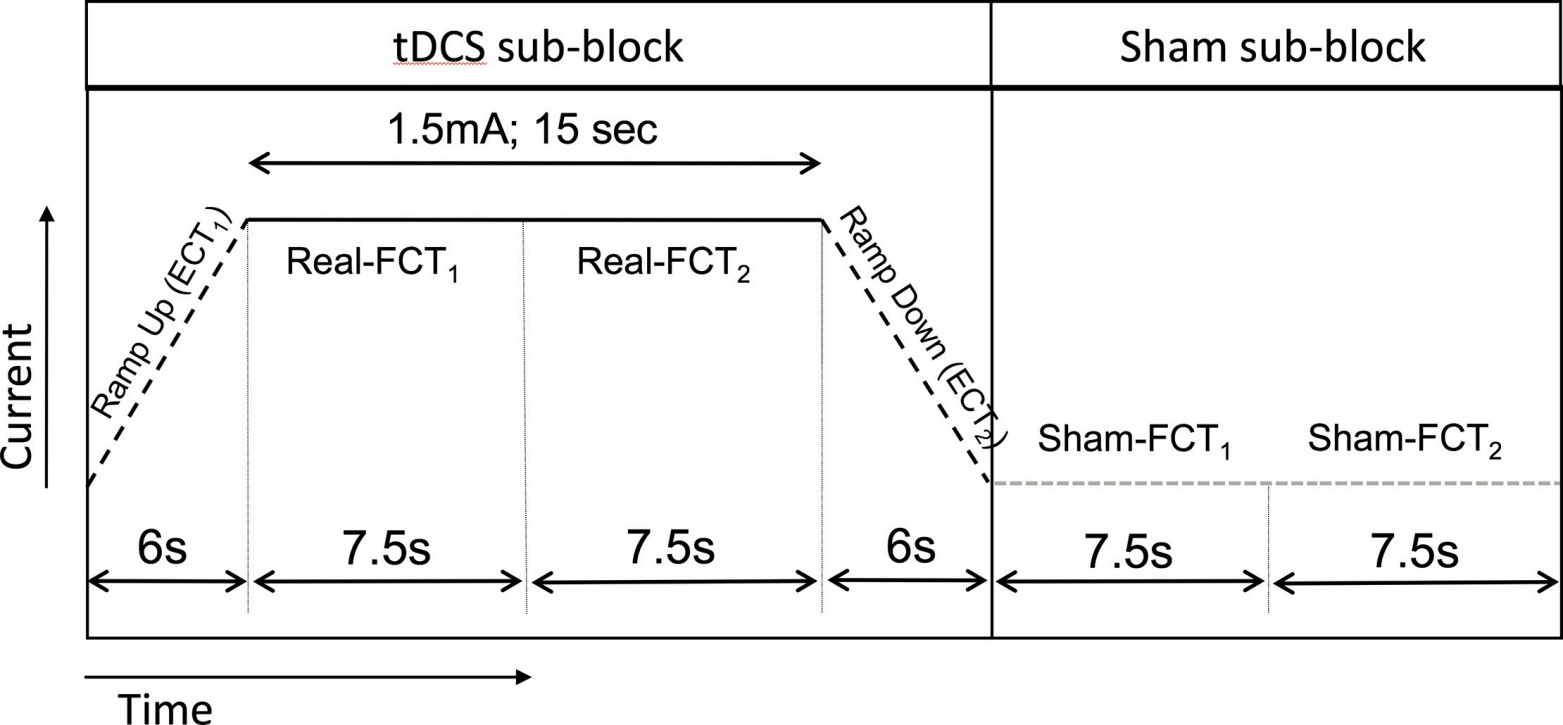
Illustration of the stimulation protocol. The tDCS sub-block included one externally-cued trial (ECT_1_), 2 consecutive free-choice trials (FCT_1_ and FCT_2_) followed by a second externally-cued trial (ECT_2_). Current was ramped up over 6s duration of ECT_1_. A constant current of 1.5mA was delivered during FCT_1_ and FCT_2_. This was followed by ramping down of the current over 6s during ECT_2_. The whole experiment consisted of 4 blocks and each block had 16 tDCS and 16 sham sub-blocks occurring in a random order. This yielded 32 free-choice trials with anodal/cathodal tDCS; 32 free-choice trials with sham stimulation and 32 externally-cued trials in every block.

We were technically limited by the minimum duration for which the stimulator would deliver current. The stimulator we used could not deliver current for a duration less than 15s. Hence, we organized every block into 16 tDCS sub-blocks and 16 sham sub-blocks. Each tDCS sub-block included 4 trials - 6s of ramping up, 15s of real anodal/cathodal tDCS stimulation and 6s of ramping down. As mentioned earlier, the ramping up and ramping down were always set to be externally-cued trials. The stimulation duration of 15s accommodated 2 free-choice trials, each lasting 7.5s. Each sham sub-block included 2 free-choice trials, each lasting 7.5s when no current was delivered. The 2 sub-blocks occurred in a completely random order. The task was programmed using Presentation® software (Neurobehavioral Systems, Berkeley, CA, USA). The software completely randomized the order of the sub-blocks and also triggered the stimulator in synchrony with the respective visual stimulus at precisely set timings. The stimulation protocol is illustrated in Fig 2. The polarities of the electrodes were changed for each experiment block and this order was also randomized across subjects. The investigators were aware of the polarity of stimulation (anodal or cathodal) in a certain experimental block. However, they did not view the display on the stimulator during the course of the experiment. Since the tDCS and sham stimulation sub-blocks occurred in a random order, they were also blinded and were not able to predict the stimulation for the upcoming trial. The subjects were unaware of both the polarity of electrodes as well as whether a specific trial involved sham or real stimulation. At the end of the experiment, all subjects reported that they felt the stimulation at about the same intensity throughout the session. Therefore, both the investigators and subjects were completely blinded to the stimulation making our results more reliable.

### Data analysis

The data from 3 subjects were not used for analysis since they did not follow the instructions correctly. They had either followed a specific sequence or had made choices depending on the previous externally cued trial. All the remaining subjects said that their choices were random and that they did not think of any specific strategy. From the EMG data of the remaining 10 subjects, we extracted the anodal, cathodal and sham free-choice trials and noted which hand was moved for each trial. We had 64 trials with anodal and 64 trials with cathodal tDCS over C3. There were also an equal number of sham trials for each polarity. The data were visually inspected for any specific pattern or sequence. Less than 5 trials per subject were discarded because the subjects either did not respond or moved both hands. Then, the preference for right hand was calculated as the ratio of the number of trials with right hand responses to the total number of valid responses and expressed as percentage.

Right hand preference = 100*RH/(RH+LH)

(RH indicates number of free-choice trials with right hand movement; LH indicates number of free-choice trials with left hand movement).

We also calculated the reaction times for each condition (anodal/cathodal/sham) for comparison. We defined reaction time as the time period from the display of the arrow on the screen to the onset of activity on the subject’s EMG recording.

IBM SPSS Statistics v22 was used for our statistical analysis. A one-way ANOVA with right hand preference as dependent factor and stimulation (3 levels: anode; cathode; sham) as independent factor was performed. Additionally, owing to the high inter-individual variability in the response to tDCS (Wiethoff et al., 2014), we also examined the hand preference at the individual subject level. We performed a Chi-square test to study the association between hand and stimulation factors. For analyzing the reaction time data, we performed a 3x2 repeated measures ANOVA with reaction time as the dependent variable and stimulation (3 levels: anode; cathode; sham) and hand (2 levels: right; left). Additionally, we also performed a 2x2 repeated measures ANOVA on sham trials to look for differences in reaction times between externally-cued and free-choice trials. We included only free-choice trials with sham stimulation for comparing reaction times since there was no constant current delivered during the externally-cued trials. For this, we used condition (2 levels: externally-cued; free choice) and hand (2 levels: right; left) as independent variables. The data were verified for sphericity and Greenhouse Geisser correction was applied wherever necessary. Post-hoc pairwise comparisons were made wherever applicable and corrected for multiple comparisons using Bonferroni method. A p value less than 0.05 was considered significant.

## Results

**Fig 3:**
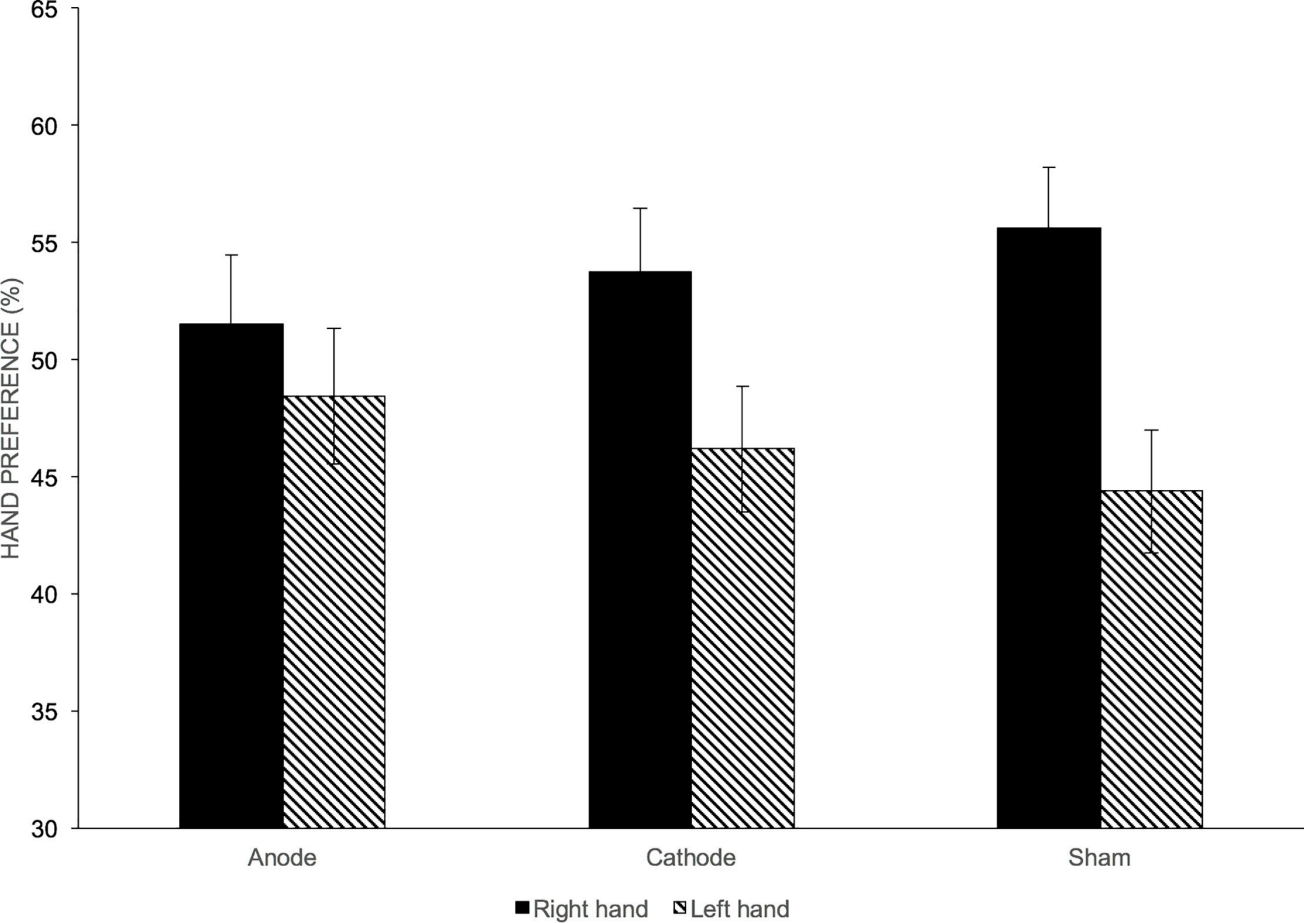
Hand preference for the different stimulation conditions. Shows the mean preference for the right/left hand in free-choice trials across subjects (expressed as percentage). The preference for the dominant right hand was strongest during sham stimulation than for real stimulation. Bars represent mean of hand preference across subjects for the different stimulation conditions. Black bars = right-hand preference; Striped bars = left-hand preference. Error bars represent standard error of mean.

**Fig 4:**
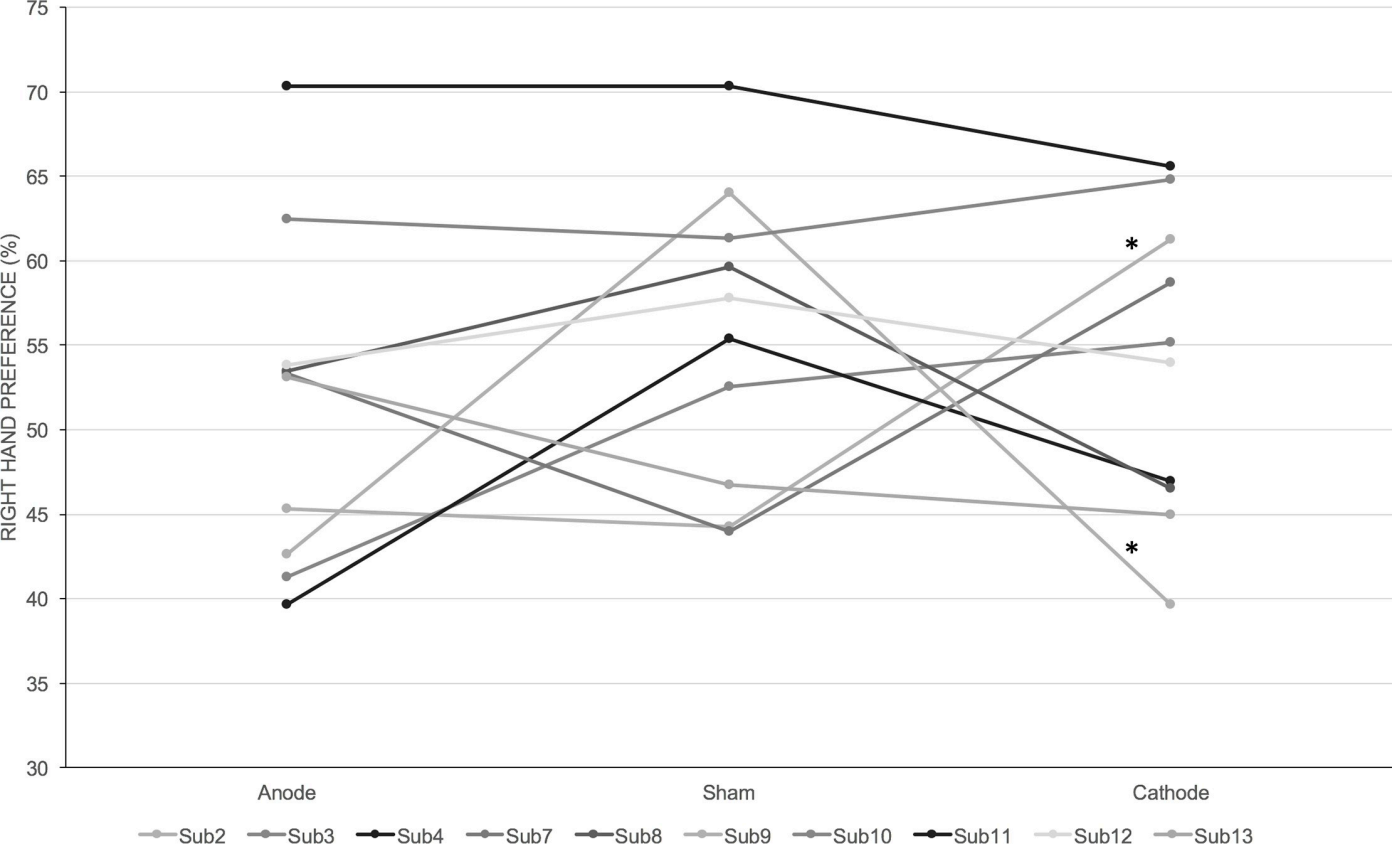
Right-hand preference in free-choice trials. Shows the right-hand preference in free-choice trials expressed as percentage for individual subjects during the different stimulation conditions. Only 2 out of 10 subjects showed significant difference in the hand preference during real vs sham stimulation (indicated by asterisks).

All subjects completed the experiment without any adverse events. One-way ANOVA did not reveal any significant effect of stimulation on right hand preference (F(2,27) = 0.495; p = 0.615). However, we observed that the subjects preferred to move their dominant right hand when we examined the sham trials alone. However, this preference did not reach statistical significance (paired t-test; t(9) = -2.023; p = 0.074). Individual subject data showed that 7 out of 10 subjects had a stronger preference for the dominant hand during sham stimulation. However, only 2 out of 10 subjects (Subject 7: X^2^(2) = 6.393; p = 0.04 and Subject 9: X^2^(2) = 10.059; p = 0.007) showed significant change in their hand preference with real tDCS. See Figs 3 and 4.

**Fig 5:**
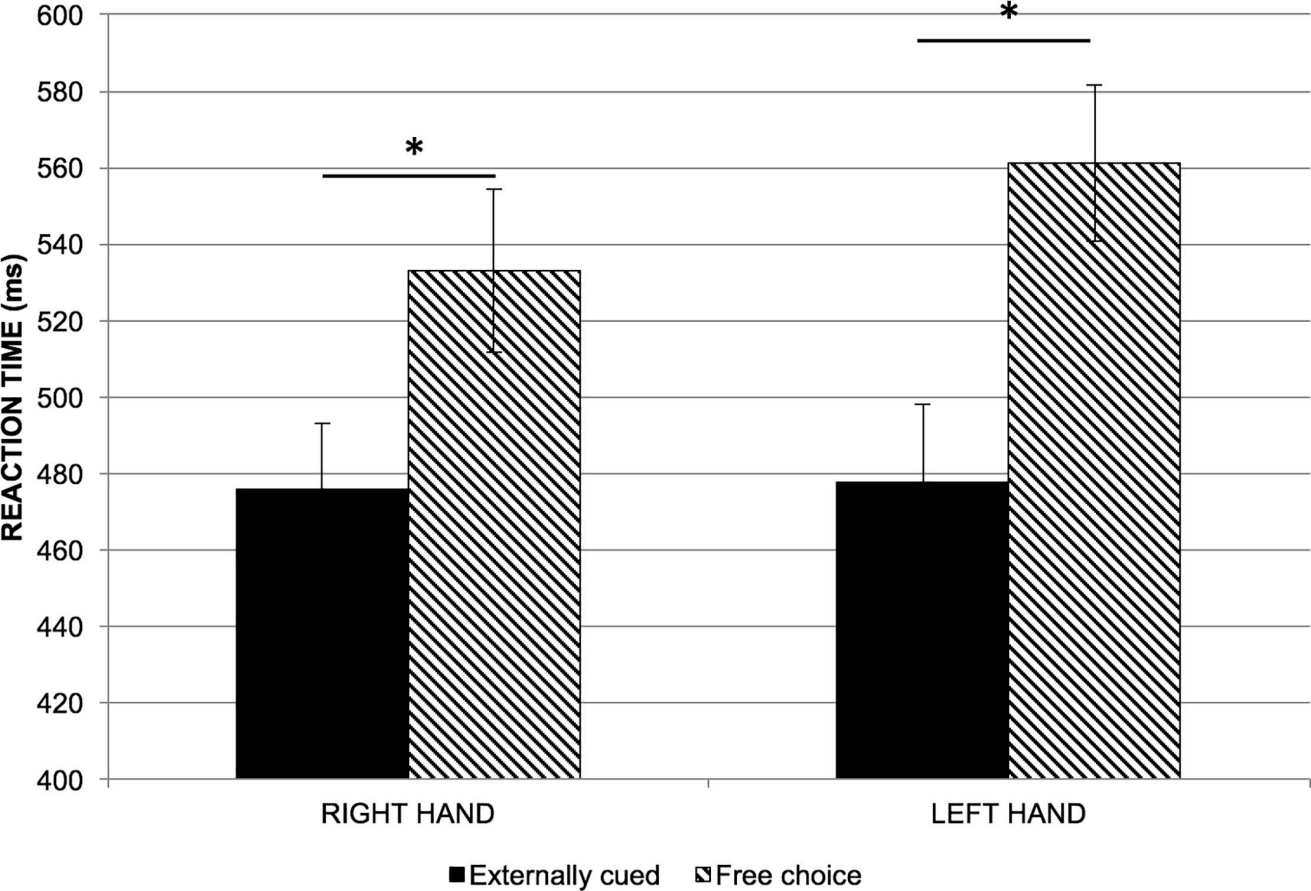
Reaction time for externally-cued versus free-choice trials. Shows the mean reaction time across subjects for the right and left hands in externally-cued and free-choice trials. Both hands had similar reaction times which was higher for free-choice trials than for externally-cued trials. Bars represent mean reaction time for each hand and condition. Black bars = externally-cued trials; Striped bars = free-choice trials. Error bars represent standard error of mean. Asterisks indicate p<0.05.

Repeated measures ANOVA on the reaction time data did not show any significant difference among the different stimulation protocols. There was neither a significant main effect of hand (F(1,9) = 1.206; p = 0.301) or stimulation (F(2,18) = 0.902; p = 0.423) nor a significant interaction effect (F(2,18) = 1.9; p = 0.178). On examining the sham trials, we found significant main effect of condition only (F(1,9) = 38.863; p<0.001). There was no significant main effect of hand (F(1,9) = 2.090; p = 0.182) or a significant interaction effect (F(1,9) = 1.415; p = 0.265). Further pairwise comparison revealed a significantly higher reaction time for free choice trials compared to externally-cued trials (p<0.001). See Fig 5.

## Discussion

In the current study, we hypothesized that modulating the motor cortical excitability using tDCS would alter movement selection. On the contrary, we found that tDCS over M1 does not influence movement selection. However, we observed a trend for preference of the dominant right hand in our subjects. We have also reported a significantly higher reaction time for free-choice trials compared to externally-cued trials.

Since M1 is the final effector in the voluntary motor pathways, it was speculated that decreasing the cortical excitability of M1 would increase the threshold for movement execution and thereby alter movement selection. Accordingly, Ammon and Gandevia [5] and Brasil-Neto et al [6] showed that single pulse TMS delivered to M1 could influence movement selection without disrupting the conscious perception of volition. Later, Sohn and colleagues [7] pointed out shortcomings in the experimental protocols and with an improved study design, failed to replicate the previous findings. They concluded that single pulse TMS over M1 was insufficient to alter movement selection. What remained to be verified was whether direct current stimulation over M1 could affect movement selection. Ammon and Gandevia [5] did not observe any effect on movement selection by stimulating M1 with 0.2–0.4mA direct current for 5s using a pair of conventional Ag-AgCl electrodes over C3 and C4. With the recently expanding literature on tDCS, we now know that this stimulation intensity and electrode size were too small to have elicited any effect [10]. Hence, we tested the same hypothesis with the current tDCS protocol and have confirmed that movement selection cannot be altered by modulating the cortical excitability of M1 using tDCS. That is, a voluntary movement cannot be inhibited/facilitated by just increasing or decreasing the cortical excitability of the final effector in the voluntary motor pathway–the M1. This indicates that intervention might be needed at a higher level to alter movement selection–probably at the motor planning areas–the premotor cortex or pre-supplementary motor area or may be even higher—the prefrontal cortex [1, 11]. Further studies are warranted to confirm this hypothesis. Several functional MRI studies have shown that internally generated movements are specifically represented in the prefrontal, premotor, posterior parietal brain regions and most importantly the anterior cingulate cortex [11, 12]. Although the association of these brain regions in movement selection and execution is well-known, a causative role is yet to be established. Non-invasive brain stimulation protocols could be helpful in determining such causal associations.

Our results also show that there is a slight bias in subjects towards choosing their dominant hand. This reiterates the strong preference that right-handed individuals have for their dominant hand [13, 14]. We also observed that this preference for the dominant hand was lost in both cathodal and anodal tDCS conditions. It is likely that the small sample size was not adequate to observe a statistically significant difference. Hence, a follow-up study with larger sample size will be needed to confirm this preliminary finding. Post hoc power analysis revealed that such a study would require a sample size of 23 to achieve a power of 80% and a level of significance of 0.05 for detecting the effect size and assuming the standard deviation observed in our study.

We also found that neither anodal nor cathodal tDCS affected the reaction time of the voluntary movement. Nevertheless, we have revealed a clearly higher reaction time of approximately 70ms involved in generating a freely chosen movement as opposed to an externally triggered movement. This longer reaction time may be attributed to the higher cognitive load associated with movement selection that involve the prefrontal and parietal regions and their basal ganglia networks [15]. This finding is consistent with our plan that in our study, the movements were more likely to be generated after a decision process in the free-choice trials.

A potential limitation of the current study is that we did not obtain an objective measure of modulation of motor cortical excitability such as motor evoked potential amplitude. Hence, the effectiveness of tDCS that was delivered may be challenged; however, we know from many previous studies that the amplitude of the current used in our experiments is effective [16]. The current was delivered for a “short” time, but there should be no delay in the presumed mechanism of modifying the membrane potential. On the other hand, we would also like to point out that although the dual-hemispheric montage has been shown to have significant after-effects on cortical excitability following several minutes of stimulation [9], there are no studies that have examined the effect of “short duration” stimulation using this montage. Further, Nitsche and Paulus [10] have shown that with this montage and with 4s stimulation given every 10s, there was no change in cortical excitability. Other confounding factors that should considered are (A) the cumulative effect of several short repetitive segments of tDCS applied within a single block and (B) the effect of consecutive or alternating blocks of anodal or cathodal tDCS during the course of the experiment. Measuring the cortical excitability before and after each block would have helped to eliminate these factors. The small sample size of the study could be another limitation but our results are very clear even at the individual subject level so there is no reason to think that a higher sample size would have helped.

In conclusion, movement selection cannot be altered by modulating the cortical excitability of the primary motor cortex. That is, it may not be possible to alter deliberative movement selection at the level of motor execution. Hence, we think that the primary motor cortex may not be the most appropriate target for therapeutic neuromodulatory protocols in patients with disorders of choice. Further studies are needed to examine if brain areas upstream of M1 in the voluntary motor pathway could be externally modulated during the process of movement selection.
